# Effect of microseparation on contact mechanics in metal‐on‐metal hip replacements—A finite element analysis

**DOI:** 10.1002/jbm.b.33313

**Published:** 2014-11-05

**Authors:** Feng Liu, Sophie Williams, John Fisher

**Affiliations:** ^1^ Institute of Medical and Biological Engineering, School of Mechanical Engineering, University of LeedsLeedsWest YorkshireUK

**Keywords:** metal‐on‐metal, hip replacement, microseparation, rim contact, finite element

## Abstract

Some early failures of metal‐on‐metal (MoM) hip replacements associated with elevated wear have caused concerns for the use of this bearing combination. Simulator studies have shown that microseparation and its associated rim contact and edge loading may produce the most severe wear in MoM bearings. It is generally recognized that this high wear can be attributed to the high contact stress of the head on the rim of the cup. In this study, an improved finite element contact model that incorporates an elastic‐perfectly plastic material property for cobalt‐chrome alloy of the metal bearing was developed in an attempt to provide an accurate prediction of the stress and strain for the rim contact. The effects of the microseparation displacement (0.1−2 mm), cup inclination angle (25−65°) and cup rim radius (0.5−4 mm) on the contact stress/strain were investigated. The results show that a translational displacement >0.1 mm under a load >0.5 kN can produce a highly concentrated contact stress at the surface of the cup rim which can lead to plastic deformation. This study also suggests that the magnitude of translational displacement was the major factor that determined the severity of the contact conditions and level of stress and strain under microseparation conditions. Future studies will address the effect of surgical translational and rotational malposition and component design on the magnitude of microseparation, contact stress and strain and severity of wear. © 2014 The Authors. Journal of Biomedical Materials Research Part B: Applied Biomaterials Published by Wiley Periodicals, Inc. J Biomed Mater Res Part B: Appl Biomater, 103B: 1312–1319, 2015.

## INTRODUCTION

Metal‐on‐metal (MoM) bearings have been used in hip joint replacements due to low wear under standard walking conditions, as shown in both laboratory simulator studies and clinical experiences.^1^, ^2^, ^3^, ^4^ MoM bearings have been further developed for surface replacement in order to preserve bone on the femoral side^5^, ^6^ and for larger head sizes to achieve enhanced range of motion and stability.^7^, ^8^ These designs were often targeted at younger, more active patients.^9^, ^10^ However, adverse conditions such as implant malpositioning can lead to edge loading or head‐cup rim contact, and produce high contact stress causing elevated wear.^11^ Recently, some early failures of MoM prostheses have been increasingly reported and caused a major concern,^12^ leading to a medical device alert issued by the Medicines and Healthcare Products Regulatory Agency in the UK.^13^

**Figure 1:**
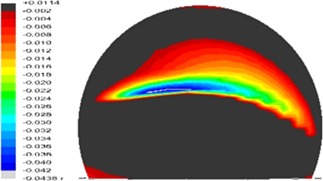
Three dimensional co‐ordinate measurement (mm) of the 36‐mm femoral head showing stripe wear due to microseparation and contact between the head and the rim of the cup obtained from a simulator test. [Color figure can be viewed in the online issue, which is available at wileyonlinelibrary.com.]

A well‐functioning hip joint implant should be able to maintain a concentric and conforming contact between the cup and head, and the contact patch thus developed should be retained well within the articulating surface during all kinds of hip kinematics.^11^ Fisher^11^ has explained that adverse conditions that can cause rim contact in hip joint replacement relate to translational and rotational malpositioning of the bearing. With rotational malposition of the cup, such as the cup being positioned in steep inclination or with excessive anteversion, the contact patch developed at the bearing surface may intersect the cup rim, and this situation can be compounded by varied bearing designs such as sub‐hemispheric cups and lower bearing clearance or by deflection of the cup bearing during fixation.^14^ Additionally, translational malpositioning can produce a more severe contact condition and edge loading.^15^ A typical condition resulting from translational malpositioning is dynamic microseparation^16^, ^17^ of hip joints or subluxation, for which a translation of the head relative to the cup occurs leading to contact at the superior lateral rim of the cup.^18^, ^19^ There are many contributing factors for microseparation including head offset deficiency, medialized cup, stem subsidence, impingement and laxity of the soft tissues.^11^ Laboratory simulator studies have shown that microseparation produces stripe wear at the bearing surfaces (Figure 1) and dramatically elevated wear rates^20^ in MoM bearings. Simulator tests have also been carried out in an attempt to investigate the effects of head sizes and cup inclination on elevated wear^20^, ^21^ under microseparation conditions; these tests have been generally focused on a fixed translation level of 0.5 mm. A full range of parametric studies which can incorporate a wide range of variations in microseparation displacement, cup orientation and cup rim radius are currently not available with simulator tests. These variables are clinically important and relevant,^22^, ^23^ and it is not known how they individually or in combination affect contact mechanics or increase wear. Finite element (FE) analysis has been developed to study stripe wear as a result of high contact stresses resulting from head‐cup rim contact associated with microseparation for ceramic‐on‐ceramic (CoC) bearings.^18^, ^24^ This method can be further developed for MoM bearings and most importantly extended into an elastic plastic contact analysis in order to predict the high stress. A systematic contact mechanics analysis has the potential to predict contact stresses and strains and provide an indication of conditions which could produce the most severe wear.

FE using commercial software has been extensively developed for contact mechanics of artificial joints.^25^, ^26^, ^27^, ^28^, ^29^, ^30^ Scifert et al.^29^ developed a contact model to study factors that can influence hip dislocation; Elkin et al.^30^, ^31^, ^32^ extended the model to investigate the effect of subluxation and impingement for MoM bearings and fracture for CoC implants; Mak et al.^18^, ^24^ calculated contact stress for edge loading due to microsepartion in CoC hip joints; Sariali et al.^33^ also carried out similar predictions for CoC bearings. Experimentally, Sanders and Brannon^34^ developed a method to predict contact area on cup edge and compared with the Hertzian contact theory. For MoM bearings, all the present contact analyses have been based on the assumption of elastic materials as a first approximation. With microseparation rim contact, the resulting high stress can exceed the yield strength of the bearing materials^32^ which requires a model that can incorporate plasticity. In addition, to capture the highly concentrated stress, a substantially fine mesh is needed to improve the accuracy of prediction.^18^

The aim of the present study was to investigate the effects of microseparation displacement, cup inclination and cup rim radius on contact stress and plastic strain in MoM bearings using an improved FE model with an elastic‐perfectly plastic model and substantially refined mesh under microseparation conditions.

## MATERIALS AND METHODS

**Figure 2:**
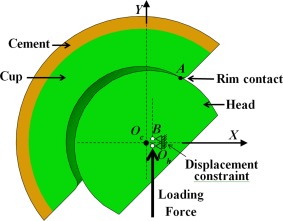
An anterior‐posterior view of the simplified half three‐dimensional contact model for MoM hip joints by considering the symmetry of the joint geometry about the medial‐lateral plane (*XY* plane) (the cup version was simplified as zero). Edge loading was modeled between the head and the superior lateral rim of the cup as a result of microseparation for which the head was translated laterally and inferiorly in *XY* plane, and then fixed at a constant lateral displacement (along *X*‐axis), and a vertical load was applied at the head centre (*O_h_*) in the *XY* plane. A node point (*B*) on the vertical axis of the head about 1 mm from the centre (*O_h_*) was also constrained. [Color figure can be viewed in the online issue, which is available at wileyonlinelibrary.com.]

A generic 36‐mm diameter MoM total hip joint bearing was analysed in this study. Both the head and cup bearings were made from wrought high‐carbon cobalt‐chrome molybdenum (CoCrMo) alloys, with the cup wall thickness of 9 mm, cup articular arc angle of 160°, and a diametric bearing clearance of 50 μm. The cup was fully bonded using polymethyl methacrylate (PMMA) bone cement at a constant thickness of 3 mm. Cup rim contact was considered as a result of a translational displacement of the head centre in the medial‐lateral plane relative to the geometric centre of the cup (Figure 2).^35^ Both the cup and head version angles were chosen to be zero to match the corresponding set‐up in a simulator test^20^ for microseparation with a focus on cup inclination only. With this simplification, a half solid geometry can be used for the computational model^25^ by making use of the symmetry about the medial‐lateral plane (Figure 2), which also allows the FE model to have a substantially finer mesh with a relatively low computational time. As the corresponding symmetric boundary, for all the nodes on the symmetry plane (Figure 2, *OXY* plane), the degree of freedom in the direction perpendicular to the symmetry plane was constrained; for the load, only a half magnitude was required and applied to the head centre. Other boundary conditions included the position of the head centre being laterally fixed (along *X*‐axis) corresponding to a given value of microseparation displacement, and the nodes on the outside surface of the cement being fully constrained. It should be pointed out that microseparation rim contact is a dynamic event,^15^, ^18^, ^35^ in which the head strikes the cup rim, and then slides on the cup rim back to the socket, and a complete analysis requires an advanced dynamic contact model. In this study, only an initial instant of the contact corresponding to the heel strike of a gait cycle was modeled and numerically solved as a quasi‐static contact^18^ between the head and cup rim rather than the full dynamic process. Both the magnitude and direction of contact force may vary depending on the external load from the ground reaction force and surrounding muscle force, the head displacement, and the duration of contact, which should be determined with the full dynamic model. Instead, in this study, the load in the vertical direction with a wide range of magnitudes from 0.5 to 3.0 kN was considered as the major loading component according to ISO 14242‐1:2002 standard for a walking cycle used with hip simulator^36^ and measured data with implanted prostheses^37^ during walking, and chosen for each of the head displacements considered. In order to implement such a static contact analysis, it is required to restrain the rotation of the head to avoid rigid body motion problem; in this study, to achieve such constraint, an adjacent node within a distance <1 mm to the head centre on the vertical axis of the head was chosen and assigned the same lateral displacement constraint along the *X*‐axis as the head centre (Figure 2).

The FE models were meshed with 8‐node brick and 6‐node triangular prism elements in NX I‐deas 6.1^25^ (Siemens PLM Software, TX). The materials were assumed to be elastic‐perfectly plastic with the yield strength of 840 MPa^38^ for the CoCr head and cup bearings, and linearly elastic for the cement, with the Young's moduli of 230 and 3.3 GPa and Poisson's ratios of 0.3 and 0.35,^39^ for the metal bearing and cement, respectively. The head‐cup rim contact was modeled with the finite sliding algorithm using ABAQUS/Implicit^40^(Version 6.11‐1; Dassault Systemes Simulia Corporation, Providence, RI). The effect of friction on the contact stress was considered as negligible^25^ in the static analysis. The contact computation for each magnitude of the vertical loads in the range of 0.5∼3kN was individually carried out without a loading history.

**Figure 3:**
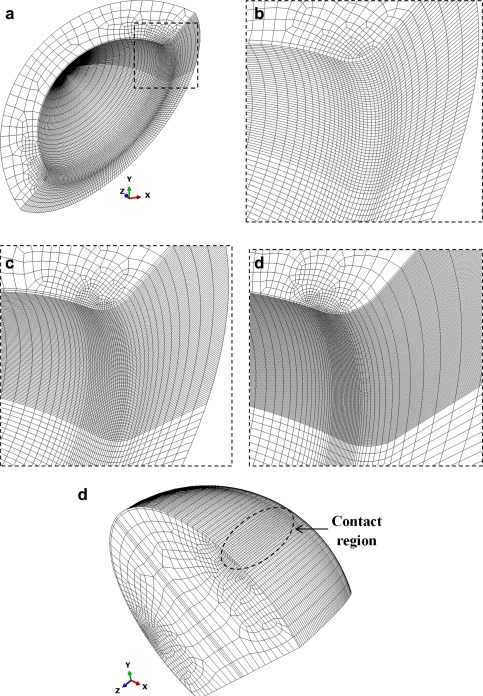
FE mesh generation and refinement: (a) the half cup model with refined mesh for the contact region as highlighted by the dashed line square, and the focused view for different mesh densities with the element sizes of (b) 0.25 mm, (c) 0.125 mm and (d) 0.0625 mm; (e) the half head model with the refined mesh of contact region as highlighted. [Color figure can be viewed in the online issue, which is available at wileyonlinelibrary.com.]

The mesh sensitivity and convergence study was conducted with a uniform mesh refinement especially designed for the local contact sites on both the cup and head [Figure 3(a–e)]. The element size of 0.25 mm was initially chosen for a coarse mesh as illustrated for the cup rim [Figure 3(b)], and repeatedly halved to 0.125 and 0.0625 mm [Figure 3(c,d)] until a minimum of 0.015625 mm. The same refinement was also made for the head to achieve a matching mesh and point‐to‐point contact between the head and cup rim, as required for the FE model to obtain accurate contact solutions.^40^ The convergence study was considered for the vertical loads of 0.5 and 3 kN, and the resulting peak contact pressures and peak equivalent plastic strains are summarized in Tables 1 and 2. A converging mesh was determined from the peak contact pressure while the plastic strain showed a relatively large discrepancy. The element size of 0.0625 mm was chosen throughout this study based on the convergence differences of 30% and 9% for the loads of 0.5 and 3 kN, respectively (Tables 1 and 2). The relatively high convergence criteria were used in this study to facilitate the parametric study. In order to achieve matching mesh at the local contact site between the head and cup rim, FE meshes were individually created for each combination of the microseparation displacement, cup inclination and cup rim radius. For the FE model with the element size of 0.0625 mm, the numbers of elements were approximately 213,000 and 3800, for the half cup and head, respectively (Figure 3). With the mesh density considered, each individual contact solution for a given load was obtained within 3−6 hours of computing time on a computer of 2.8 GHz, 12Gb RAM.

**Table 1:** FE Mesh Convergence Check Based on the Cup of 2‐mm Rim Radius in 45° Inclination Under the Vertical Loading Force of 0.5 kN

Element Size (mm)	Maximum Contact Pressure (GPa) and Difference Percentage		Maximum Plastic Stain and Difference Percentage	
0.25	0.53	–	–	–
0.125	0.99	46%	0.001	–
0.0625	1.41	30%	0.0039	74%
0.03125	1.84	23%	0.0037	5%
0.015625	2.04	10%	0.008	54%

**Table 2:** FE Mesh Convergence Check Based on the Cup of 2‐mm Rim Radius in 45 Inclination Under the Vertical Loading Force of 3 kN

Element Size (mm)	Maximum Contact Pressure (GPa) and Difference Percentage		Maximum Plastic Stain and Difference Percentage	
0.25	1.40	–	0.007	–
0.125	1.82	23%	0.0087	20%
0.0625	2.00	9%	0.0094	7%

Three sets of conditions were analyzed: (1) the effect of head lateral translation varying from 0.1 to 2 mm for the cup with a rim radius of 2 mm at a fixed cup inclination of 45°; (2) the effect of cup inclination increasing from 25° to 65° for the cup with a rim radius of 2 mm at a fixed head translation of 0.5 mm; (3) the effect of cup rim radius varied at 0.5, 2, and 4 mm for a cup inclination of 45° and head translation of 0.5 mm.

## RESULTS

**Figure 4:**
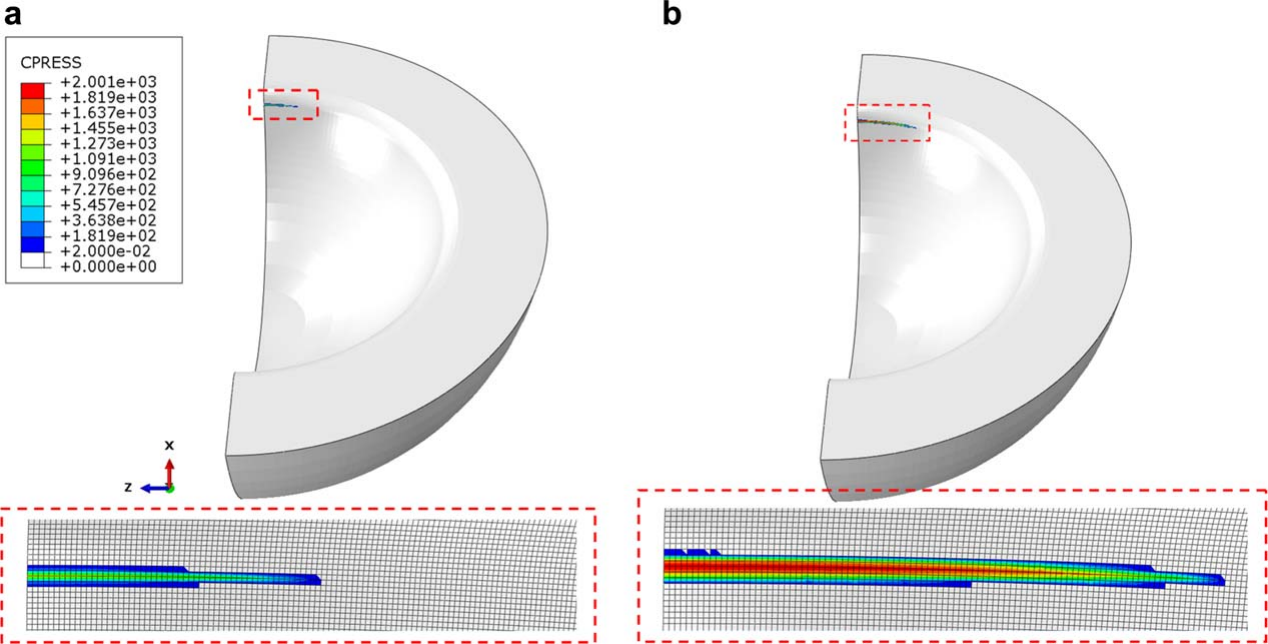
Contact pressures predicted at the cup rim, inferiorly viewed (along the *Y* axis), and the detailed distributions on the FE mesh (element size of 0.0625 mm) for the cup of 2 mm rim radius in 45° inclination with the head displacement at 0.5 mm, under the vertical loading forces of (a) 0.5 kN and (b) 3.0 kN; the contact lengths for the half model being 3.44 and 6.25 mm and the contact widths being 0.25 and 0.38 mm corresponding to the above two loads, respectively. [Color figure can be viewed in the online issue, which is available at wileyonlinelibrary.com.]

With the improved FE model, which included an elastic‐perfectly plastic material property for CoCr in MoM bearings for the first time and a convergent mesh to address high stress concentrations, improved predictions of contact stress and stress distribution were obtained for MoM bearing under conditions of microseparation rim contact. A representative contact pressure distribution at the cup rim theoretically predicted with the half contact model is shown in Figure 4, where the cup has a 2 mm rim radius, 45° of inclination and 2 mm of head displacement. An approximately elliptical contact patch was predicted at the superior lateral rim of the cup, with the maximum contact lengths being 7−13 mm (over the full cup rim) and the maximum contact widths 0.3−0.4 mm for the loads of 0.5−3 kN.

**Figure 5:**
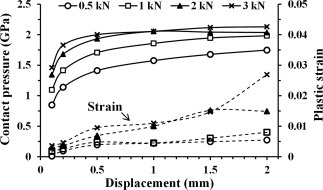
Effect of head displacement, 0.1–2 mm, on the maximum contact pressure and the maximum equivalent plastic strain at the cup rim, for the cup with 2 mm rim radius in 45° inclination, under varying vertical loading forces between 0.5 and 3 kN. The resultant contact forces on the head slightly varied from the vertical loading forces applied, due to a contribution from the head displacement constraint along the medial‐lateral direction, for example, in the ranges from 575 to 546 N (variation < 4%) and from 3435 to 3275 N (variation < 5%) corresponding to the vertical loading forces of 0.5 and 3 kN, respectively, over the head displacements from 0.1 to 2 mm.

For the head displacement at 0.1 mm with the load at 500 N, the peak contact stress (von Mises stress) at the cup rim exceeded the yield strength 840 MPa of wrought CoCr alloy and plastic deformation occurred as shown in Figure 5. Generally, both the peak contact pressures and peak plastic strains increased with the increasing head displacement for a constant load, and increased with the increasing load for a constant head displacement, over the range of 0.1−2 mm for head displacement and 0.5−3 kN for load (Figure 5). The peak contact pressure in particular increased steeply over the lower displacements (<0.5 mm), for example, by 65% (from 0.83 to 1.38 GPa) for the head displacement varying from 0.1 to 0.5 mm (the load at 0.5 kN) compared with 27% (from 1.38 to 1.75 GPa) for the head displacement being further increased from 0.5 to 2 mm.

**Figure 6:**
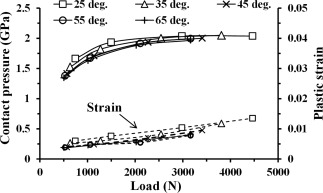
Effect of cup inclination angle, 25–65°, on the maximum contact pressure and the maximum equivalent plastic strain at the cup rim, for the cup of 2 mm rim radius with the head displacement at 0.5 mm, under varying vertical loading forces between 0.5 and 3 kN. The resultant contact forces were chosen as the variable for the horizontal axis to include a contribution from the head displacement constraint along the medial‐lateral direction. The resultant contact forces were 0.745−4.464 kN with the largest variation (50%) for the cup in 25° inclination, compared to the vertical loading forces 0.5−3 kN, at the head displacement of 0.5 mm.

**Figure 7:**
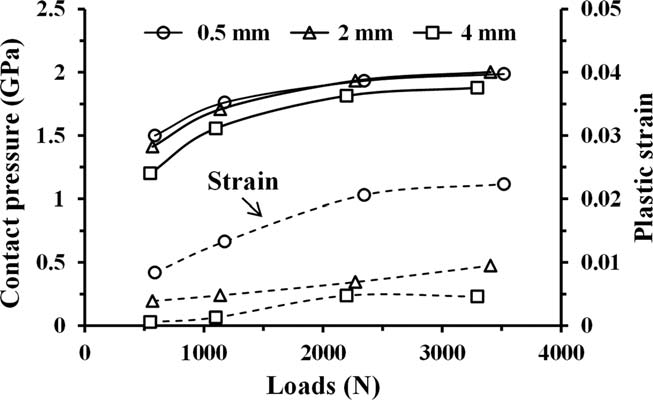
Effect of cup rim radius, 0.5–4 mm, on the maximum contact pressure and the maximum equivalent plastic strain at the cup rim, for the cup in 45° of inclination with the head displacement at 0.5 mm, under varying vertical loading forces between 0.5 and 3 kN. The resultant contact forces (5% larger than the corresponding vertical loading force for the 2‐mm cup rim radius case) were chosen as the variable for the horizontal axis to include a contribution from the head displacement constraint along the medial‐lateral direction.

The effects of varying cup inclinations and varying cup rim radii on both the peak contact pressure and plastic strain are respectively shown in Figures 6 and 7. For the cup with a 2‐mm rim radius and the head displacement at 0.5 mm at any given constant load (Figure 6), increasing inclination angles from 25 to 65° led to both slightly decreased contact pressures and plastic strains, by approximately <15% and <30%, respectively. In Figure 7, for the cup in a constant 45° inclination with a constant head displacement at 0.5 mm at any given constant load, increasing cup rim radius led to both decreased contact pressure and plastic strain. In particular, the decrease for plastic strain was larger compared with contact pressure, for example, approximately 50 and 6%, respectively, at a constant load of 3 kN (Figure 7).

## DISCUSSION

Currently, direct measurement of contact stress requires use of pressure sensors.^41^ However, with the challenges associated with a highly conforming joint and limited resolution of sensors, this method is generally not suitable for a contact with highly concentrated stress resulting from rim contact. Alternatively, Sanders and Brannon^34^ developed a fingerprinting technique to provide a measurement of contact dimension for rim contact. In this experimental study, ceramic‐on‐metal hip bearings were reported, and the measured contact dimensions were found to be larger, compared with the prediction obtained from the Hertzian contact theory, for example, by 5–25% and 10–65% for the major and minor axes of the contact distribution under a load range of 300–3000 N, respectively. This study clearly showed that plastic deformation occurred in the metal bearing. Importantly, it also indicates that an improved finite element model is necessary to incorporate plasticity and finer mesh to capture the highly concentrated stress. The large contact stress that exceeds the material yield strength of metal bearings as a result of rim contact has also been confirmed in another finite element study.^32^

A physical simulation of a dynamic microseparation has been carried out using hip simulators, which has been demonstrated to show comparable wear patterns to that observed on retrievals.^15^ Computational models using motion and load inputs determined from kinematic and inverse dynamic data from human‐subject optoelectronic motion capture can provide an alternative approach to the physical simulation.^32^ A fluoroscopic technique has been developed to detect the translational displacement of the cup and head bearings under dynamic conditions.^16^, ^17^ However, with the complexity of dynamic microseparation, there is no *in vivo* dynamic data available with respect to the contact loads and kinematics associated with microseparation and corresponding rim contact. The use of a computational model based on the gait cycle condition used by hip simulator was deemed to be a reasonable approximation for the microseparation simulation.

Microseparation of the head relative to the geometric centre of the cup in MoM bearings during a walking cycle can produce a severe contact between the head and the superior lateral rim of the cup.^15^ This contact leads to a substantially narrowed contact area (Figure 4) particularly along the minor axis of the contact patch (contact width, 0.25−0.38 mm) and highly concentrated contact stress (contact pressure) resulting in local stresses beyond the yield strength of CoCr alloy and a permanent deformation at the rim. The computed contact dimensions were generally comparable with an experimental measurements,^34^ in which a 36‐mm ceramic‐on‐metal (CoM) bearing showed the major and minor axes being, respectively, in the ranges of 4−6 mm and 0.3−0.44 mm, for the load range of 0.6−2.5 kN. The maximum contact length presently predicted for the MoM bearing (7−13 mm) was larger than the measured contact length (4−6 mm) of CoM bearing, and this difference can be attributed to the higher Young's modulus 380 GPa of the ceramic head compared with 230 GPa for the metal head of the present FE model. The present model in particular shows that microseparation rim contact with a lower displacement of 0.1 mm at a lower load of 0.5 kN produced high stress leading to yielding of the material in MoM bearings (Figure 5). To accurately predict the stress concentration and plastic strain, the present FE analysis required the element size < 0.06 mm for contact surfaces to attain a converged contact solution. This element size is approximately 10 times lower than the 0.5 mm used for the elastic model as obtained from the previous FE studies.^32^ This result demonstrates that both an elastoplastic model and a substantially fine FE mesh are needed for the rim contact model under microseparation conditions.

Previous studies have shown that MoM hip joint bearings under standard walking conditions generally produce contact pressures <100 MPa^26^, ^27^, ^42^ and wear rates <1 mm^3^ /million cycles.^11^ It has been found that such standard conditions can develop a mixed lubrication mode w ith some fluid film support and a layer of lubricant protein and metallic nanoparticles acting as solid lubricant to sustain a mild level of wear.^43^ In contrast, microseparation rim contact produce stripe wear which has been associated with both fatigue and abrasive wear with elongated pits and scratches causing significantly rougher wear surfaces, and the wear rate being 15 times higher for microseparation at 0.5 mm.^20^ The present theoretical prediction clearly quantifies the high stress condition of stripe wear for MoM bearings. For example, the peak contact pressure generally ranged from 850 MPa to 2 GPa for the head displacement in the range of 0.1−2.0 mm and the load in the range of 0.5−3 kN (Figures 5, 6, 7). The average contact pressure over the theoretically computed contact area, especially for a representative cup of 2 mm rim radius in 45° inclination at the low load of 0.5 kN, ranged from 385 to 631 MPa over the displacements of 0.1−0.5 mm, and increased to 905 MPa as the displacement increased to 2 mm (Figure 5). Under such an extreme loading, the protein tribolayer is absent in MoM bearings under severe stress conditions.^44^ The present results also indicate the dependency of the elevated wear on the increase in contact pressure resulting from microseparation rim contact and increasing head displacements.

Among these factors considered in this study, microseparation displacement was the most influential variable that determined the location of contact relative to the cup rim and consequently variations in contact stress and strain. As head displacement increased, the contact point was displaced laterally towards the lateral side of the rim (moving along the circle of the rim in the cross‐sectional plane *OXY*). This lateralization of the contact site led to the conformity of contact and the rigidity of the contact site being reduced; as a result, both the contact stress and plastic strain increased. For example, when the head displacement increased from 0.1 to 2 mm, the peak contact pressure increased by 112% (from 0.85 to 1.8 GPa for the load of 0.5 kN), and the peak plastic strain increased by 25‐fold (from 0.0002 to 0.005) (Figure 5). In particular, the pressure increase was rapid (∼65%) over the lower displacements (0.1−0.5 mm) compared with the increase (∼27%) over the higher displacements (0.5−2 mm) at a load of 0.5 kN (Figure 5). This markedly varied contact pressure for the lower displacements <0.5 mm may in part explain the relatively large variations in wear rates measured with simulator tests for microseparation at 0.5 mm.^20^

Conversely, both increasing cup inclination and increasing cup rim radius led to the location of contact patch at the cup rim being slightly displaced medially towards the boundary of the rim at the cup articular surface. With the contact site being closer to the cup bearing surface, both the conformity and rigidity of the contact region at the rim were increased, and hence both contact pressure and plastic strain were reduced (Figures 6 and 7). However, for a constant head displacement at 0.5 mm with a given load, the effect of both varying cup inclination (25−65°) and varying cup rim radius (0.5−2 mm) on the location of contact was generally small (<15 and <6%, respectively); the contact patches were found to be constantly distributed within the rim surface, and therefore both contact pressures were consistently high (Figures 6 and 7). This may further indicate that the effect of varying cup inclination or cup rim radius on microseparation wear can be less great for the head displacement being kept constant. This tendency for cup inclination is consistent with the simulator results,^20^ of which the wear rate for 36‐mm MoM bearings with microseparation at 0.5 mm was 5.47 mm^3^/million cycles for the cup in 45° inclination and 4.14 mm^3^/million cycles (24% decrease) as the cup inclination increased to 65°.

This study has some limitations. The contact force between the head and cup rim has been currently considered in a range of 0.5−3 kN. Both the direction and magnitude can be varied depending on various parameters such as microseparation displacement, the duration of rim contact, and external forces including both the ground reaction and muscle forces. A full analysis of the dynamic contact is required with an advanced dynamic contact model to incorporate all the factors involved for which a preliminary dynamic model is to be reported.^45^ The cup version angle was not included which may complicate the variation in contact location at the cup rim and lead to varied trends in contact stress as indicated in the elastic model study.^32^ The present elastic‐perfectly plastic model is an improved approximation of the material properties of CoCr in MoM bearings, and the contact modeling was based on the original bearing geometry without considering the loading history. Both the permanent deformation and bearing surface wear can change the rim geometry and therefore influence the contact stress. All these issues indicate that the computational model needs to be further improved to compliment simulator studies and to address complicated clinical variability. This study has controlled the translation of the centers of the head and cup as an input to the contact model and shown that the magnitude of translation during dynamic microseparation is a critical factor in determining the severity of the contact during edge loading. Future studies will investigate how the combination of factors such as level of surgical mal‐positioning, both translational and rotational, cup design and biomechanical inputs influence the amplitude of dynamic microseparation translation and hence the severity of the edge contact.

Microseparation during a gait cycle leads to rim contact between the head and the superior lateral rim of the cup, and causes severe stress concentrations and substantially high contact stress, which produces elevated wear. The high contact stress critically depends on the translational displacement of the head bearing occurring relative to the centre of rotation of the cup. It is suggested that both the design and surgical factors of all hip joint bearings should be considered to avoid such a malpositioning adverse condition to ensure low wear performance.
